# The effects of microchipping C57BL/6N mice on standard phenotyping tests

**DOI:** 10.12688/f1000research.21633.2

**Published:** 2020-04-06

**Authors:** R.S. Bains, H.L. Cater, M. Stewart, C.L. Scudamore, S.E. Wells

**Affiliations:** 1 MRC Harwell Institute, Mary Lyon Centre, Harwell Campus, Oxfordshire, OX11 0RD, UK

**Keywords:** RFID, Microchips, phenotyping, C57BL/6NTac, mice, Home cage analysis, histopathology

## Abstract

The C57BL/6N inbred lines of mice are widely used in genetic research. They are particularly favoured in large scale studies such as the International Mouse Phenotyping Consortium (IMPC), where C57BL/6N mice are genetically altered to generate a collection of null alleles (currently more than 8500 null alleles have been generated). In this project, mice carrying null alleles are subjected to a pipeline of broad-based phenotyping tests to produce wide ranging phenotyping data on each model. We have previously described the development of a Home Cage Analysis system that automatically tracks the activity of group housed mice from a microchip inserted in the groin. This platform allows assessment of multiple biologically relevant phenotypes over long periods of time without experimenter interference, and therefore is particularly suited for high through-put studies. To investigate the impact of microchips on other tests carried out in the IMPC pipeline, we inserted microchips in 12 male and 12 female C57BL/6Ntac mice at seven weeks of age. Starting at nine weeks of age these mice underwent standard phenotyping tests, concurrently with 20 unchipped C57BL/6Ntac mice (10 females, 10 males). Tissues from a subset of the microchipped mice (six males and six females), chosen at random, were also sent for histopathological examination at the end of the phenotyping pipeline. No significant impact of insertion of microchip was observed in any of the phenotyping tests apart from bone mineral density measurement at DEXA due to the nature of the microchip. We therefore recommend that the microchip be inserted during the DEXA procedure, after the measurement is taken but before the mouse has recovered from the anaesthetic. This would avoid multiple anaesthetic exposures and prevent the potential variability in DEXA analysis output.

## Introduction

The overarching aim of phenotyping studies is to understand and describe the relationship between genotype and phenotype. However, mouse behaviour is variable, dynamic and adaptive and therefore is influenced by a variety of genetic and environmental factors such as motivation, interaction with the experimenter, experimental design, test order, testing time and environmental enrichment ^1, 2^. In recent years, automated home cage phenotyping as a complement to conventional out-of-cage testing has increasingly been used to enrich phenotype datasets ^3^. We have been developing a novel Home Cage Analysis system (HCA) for multiply housed mice that is entirely compatible with the modern high density individually ventilated caging (IVC) systems. The HCA allows us to record automated detailed behavioural parameters over time in an undisturbed standard rack-mounted cage - the type the mice are born, reared and constantly housed in - within their established social groups. The HCA combines radio frequency identifier (RFID) tracking with video recording for automated behavioural scoring. For optimal resolution of the RFID signals from the microchips, the chips are inserted subcutaneously in the animal’s groin ^4^. Though designed to investigate subtle phenotypes in undisturbed animals, thereby reducing variability, it is possible that the insertion of the RFID microchip may itself influence behaviour, thus changing the phenotyping profile. 

Implantable micro-identification has been used as an effective means of identification in long term rodent studies for over two decades; where a hermetically sealed inert glass cylinder, 12mm in length and 2mm in diameter, containing the microchip transponder is implanted subcutaneously in the mid dorsal region of the animal ^5, 6^. Although widely considered safe, some long term studies have reported the development of foreign-body-induced tumours in various mouse strains ^7, 8^. Furthermore, the subcutaneous implantation of the microchip in the groin requires the mice to be anaesthetised; although gaseous anaesthesia is brief (less than one minute), this may interfere with the phenotyping tests further downstream ^9^. The implant itself may also confound certain assays such as dual-energy X-ray absorptiometry (DEXA) which measures total body composition. The current study investigates phenotypic effects of subcutaneous microchip implants in the groin of C57BL/6NTac mice. At the end of the study the tissue around the microchip was sent for histopathology to detect any signs of foreign-body induced pathology.

## Methods

### Ethics statement

All procedures and animal studies were carried out in accordance with the Animals (Scientific Procedures) Act 1986, UK, Amendment Regulations 2012 (SI 4 2012/3039). This study has been approved by the Animal Welfare and Ethical Review Board (AWERB), resulting in licence 30/2890. All efforts were made to minimize suffering by considerate housing and husbandry, the details of which are available at the IMPC portal: http://www.mousephenotype.org/about-impc/arrive-guidelines. Animal welfare was assessed routinely for all mice involved.

Adult mice were killed by terminal anaesthesia followed by exsanguination, and death was confirmed by either cervical dislocation or cessation of circulation.

### Husbandry

22 C57BL/6NTac male and 22 C57BL/6NTac female mice, bred at the Mary Lyon Centre, Harwell, were housed in IVC’s, on Eco-pure spruce chips grade 6 bedding (Datesand, U.K.), with shredded paper shaving nesting material and small cardboard play tunnels for enrichment. Mice were randomly assigned to groups of three to five mice per cage and the phenotyping tests were carried out as a part of a bigger batch of experimental animals and their position in the batch was randomly allocated. The method of randomisation used was Simple Randomisation, and the experimenters were blinded to the test groups. Only the experimenters carrying out X-ray analysis after the test would know the groups as that would not be possible to blind. The mice were kept under controlled light (light 7 a.m. to 7 p.m., dark 7 p.m. to 7 a.m.), temperature (21 °C ± 2 °C) and humidity (55% ± 10%) conditions. They had free access to water (25 p.p.m. chlorine) and were fed *ad libitum* on a commercial diet (SDS Rat and Mouse No.3 Breeding diet (RM3)).

Animal welfare checks were carried out visually once a day.

### Microchipping

The microchipping procedure has been described in Bains *et al.,* 2016 ^4^. Briefly, RFID microchips were injected subcutaneously into the lower left or right quadrant of the abdomen of each mouse at seven weeks of age. These microchips were contained in standard ISO biocompatible glass capsules (11.5×2mm, PeddyMark Ltd. UK). The procedure was performed on anaesthetised mice (Isoflo, Abbott, UK) after topical application of local anaesthetic cream on the injection site prior to the procedure (EMLA Cream 5%, AstraZeneca, UK). While using local anaesthetic is not common practice, we have found that a combination of these anaesthetics is the most refined method of delivering these scientific data, as this minimizes any acute discomfort the animal may feel after recovering from the general anaesthetic.

No sutures were required.

### Phenotyping pipeline

12 male and 12 female C57BL/6NTac mice were microchipped in the groin at seven weeks of age and phenotyped alongside 10 male and 10 female C57BL/6NTac baseline mice (n=24-20), starting at nine weeks of age. 20 baseline and 24 microchipped mice underwent systematic broad-based phenotyping for adults, according to the pipeline described in Table 1. The reason for the difference in numbers in each experimental group was due to the HCA being optimised for three animals in a home cage while the mice in the standard phenotyping pipeline are housed in groups of five. A sample size of 10 mice per sex was calculated based on an alpha of 1% and power of 0.8. All standard operating procedures have been described within the International Mouse Phenotyping Resource of Standardised Screens, the IMPReSS database ( www.mousephenotype.org/impress).

**Table 1.** Phenotyping pipeline

Test	Phenotypic area	Age (weeks)
Body weight	General health and welfare/essential for certain tests	Weekly from 4 to 16 weeks
Open Field	Anxiety and exploratory behaviours	9
Combined SHIRPA and Dysmorphology (CSD)	Neurological/gross morphology	9
Grip Strength	Musculoskeletal/neurological	9
Acoustic Startle and Pre-pulse Inhibition	Sensorimotor gating	10
IPGTT	Metabolic	13
X-ray	Musculoskeletal	14
Body Composition (DEXA lean/fat)	Musculoskeletal and body composition	14
Auditory Brain Stem Response + Click Stimulus	Hearing	14
Slit Lamp/Opthalmoscope	Vision/eye morphology	15
Terminal	Haematology Clinical blood chemistry Heart weight Gross Pathology and Tissue Collection Tissue embedding and Histopathology from block, where required	16

The table outlines the phenotyping tests in the pipeline in the order that they are carried out in, along with the age in weeks that each test is conducted. SHIRPA, SmithKline Beecham, Harwell, Imperial College and Royal London Hospital phenotype assessment; IPGTT, intraperitoneal glucose tolerance test; DEXA, dual energy X-ray analysis.

To provide traceability, IMPReSS also stores a record of change histories, all tests in this study were carried out at the Mary Lyon Centre, MRC Harwell Institute, between November 2015 and March 2016.

### Necropsy and pathology

At the end of the phenotyping pipeline, six randomly chosen microchipped males and six randomly chosen microchipped females at 16 weeks of age underwent necropsy. The animals were simultaneously randomised without considering any variable as a part of a larger batch of animals undergoing the same procedure. The choice of euthanasia, overdose of anaesthetic followed by exsanguination, was considered the most refined and appropriate for gathering the data for clinical chemistry and haematology assays by the AWERB. The site of microchip implantation was examined and the tissue surrounding the implant was fixed in 10% neutral buffered formalin (NBF). Tissue samples from the implant sites were processed routinely to wax and 3µm sections were stained with haematoxylin and eosin (H&E) and examined by a veterinary pathologist.

### Data analysis

Data for Open Field test, Grip Strength test, Acoustic Startle and Pre-pulse Inhibition and DEXA were analysed using the Student’s t-test with Welch’s correction for unequal variance. The data for weekly weight curve and intraperitoneal glucose tolerance test (IPGTT) were assessed using two-way analysis of variance (ANOVA) with Sidak post hoc analysis. As the current study tests multiple hypotheses, Bonferroni’s correction was applied to avoid type 1 error in the interpretation of the data. Therefore, an alpha value of < 0.001 was calculated based on an alpha of 0.01 and R=11 tests, for an effect to have statistical significance. The categorical data such as Combined SHIRPA and Dysmorphology (CSD), opthalmology and X-ray were analysed using Fisher’s exact test. GraphPad Prism 8.3.0 was used for statistical analysis.

## Results

### Phenotyping

***Dysmorphology.*** No evidence of any major differences in morphological features was found between the chipped and unchipped mice. The X-ray analysis showed that microchips were situated at the site of implantation.

**Figure 1.**
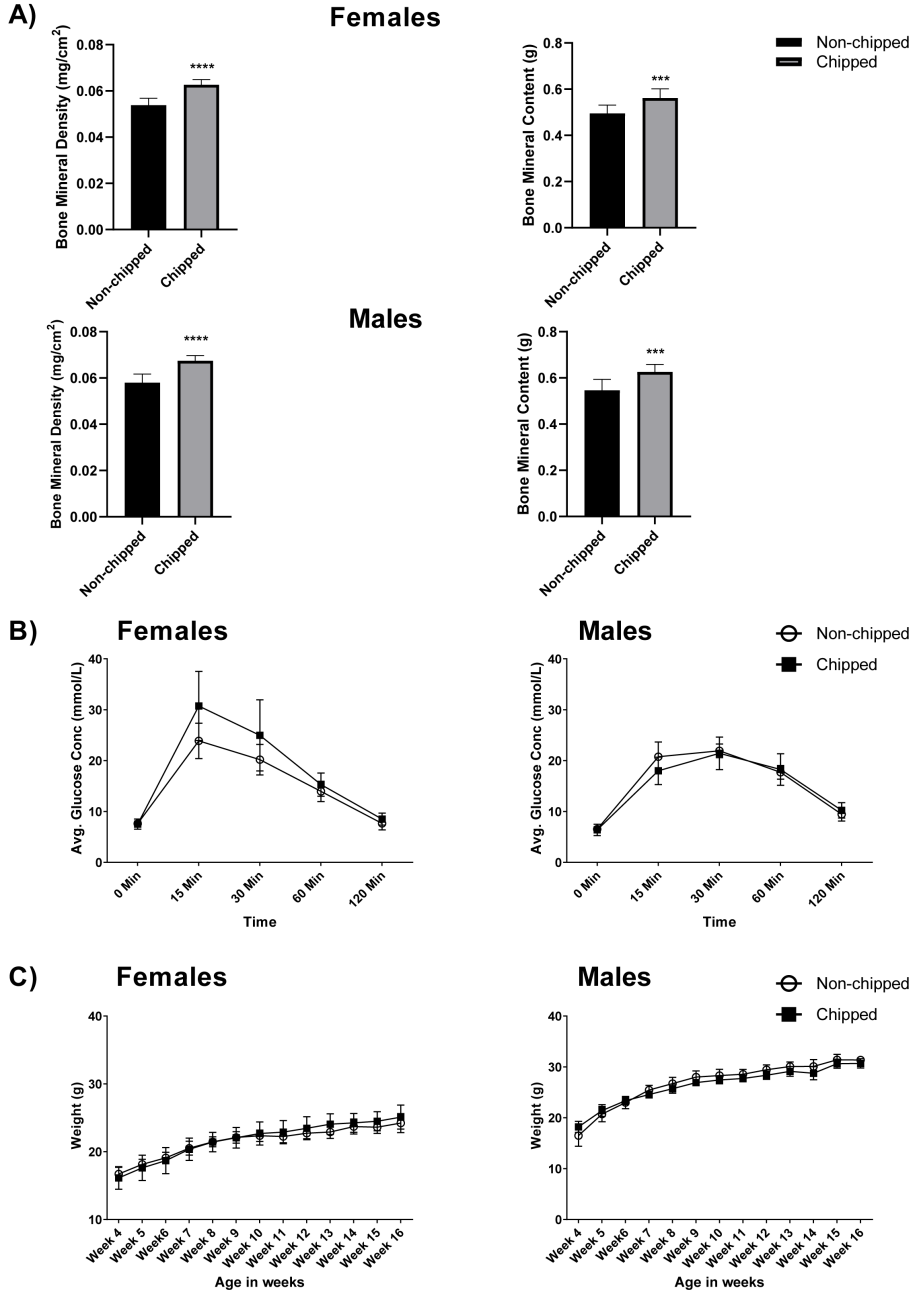
**A**) Bars represent average bone mineral density (g/cm ^2^) and average bone mineral content (g), for males and females as measured through dual-energy X-ray absorptiometry (DEXA). Microchipped females as well as males show a significant increase in the two parameters as compared to unchipped animals (n=10-12; ***p<0.001 and ****p<0.0001), the T bars represent ±sem. **B**) The lines represent average glucose concentration (mmol/L) over time as measured through IPGTT, the shapes represent average glucose concentration (mmol/L) for each group at that time point, the T bars represent ±sem. No statistically significant difference was seen in either sex for microchipped versus unchipped mice. **C**) The lines represent the body weight curve of the two treatment groups over time, the shapes represent mean body weight (gm) for each group at that time point, the T bars represent ±sem. No statistically significant difference was seen in either sex for microchipped versus unchipped mice.

***Metabolism.*** There was no significant difference between the weight curve data of chipped mice compared to unchipped mice over the 12-week testing period ^10^. Figure 1C represents the mean body weight±sem of each group compared every week starting at four weeks of age (16.71±0.29 non-chipped vs 16.13±0.46 chipped females; 16.5±0.63 non-chipped vs 18.22±0.29 chipped males), until the end of the study at 16 weeks (24.22±0.27 non-chipped vs 25.1±0.5 chipped for females; 31.37±0.19 non-chipped vs 30.7±0.41 chipped males). DEXA body composition measurements, represented in Figure 1A, showed significant differences between the two groups, when analyzing bone mineral density (p<0.0001; mean±sem= 0.054±0.001 non-chipped vs 0.063±0.001 chipped females; 0.057±0.001 non-chipped vs 0.067±0.001 chipped males) and bone mineral content (p<0.001; 0.49±0.01 non-chipped vs 0.56±0.01 chipped females; 0.55±0.01 non-chipped vs 0.63±0.01 chipped males) in both males and females. This can be attributed to the texture of the microchip, which would behave similarly to bone when imaged using DEXA. The results from the IPGTT, represented in Figure 1B, showed an increase in mean blood glucose concentration at the 15-minute time point for microchipped females (30.73±1.87) versus unchipped females (23.88±1.04), but this was not statistically significant. No evidence of altered glucose metabolism was seen in chipped males (17.99±0.87) versus unchipped males (20.76±0.86) for the 15-minute time point.

***Neurological, behavioral and sensory.*** No significant differences were observed in the whole arena open field locomotor parameters between chipped and unchipped mice (means±sem= 12.08±0.40 for non-chipped vs 12.32±0.36 chipped females; means±sem=12.06±0.51 for non-chipped vs 10.73±0.19 chipped for males); this was also true for the locomotor activity observed in the arena during the modified SHIRPA testing, where all animals tested showed normal activity. Grip strength measurements of fore and hind limbs normalized to body weights between the two groups were also non-significant (means±sem=4.89±0.19 for non-chipped vs 5.12±0.19 chipped females; means±sem=4.91±0.21 for non-chipped vs 4.83±0.24 chipped for males).

Acoustic startle (means±sem of startle amplitude= 481.75±29.86 non-chipped vs 443.79±31.19 for females; 668.33±53.71 non-chipped vs 499.27±50.39 chipped males) and startle response to pre-pulse showed no significant difference between the chipped and unchipped mice (means±sem of pre pulse1 startle amplitude= 530.64±48.46 non-chipped vs 418.6±23.9 for females; 659.28±57.03 non-chipped vs 504.65±42.24 chipped males; means±sem of pre pulse2 startle amplitude= 298.03±19.76 non-chipped vs 254.7±18.96 for females; 315.35±32.48 non-chipped vs 275.58±32.29 chipped males; means±sem of pre pulse3 startle amplitude= 302.53±18.37 non-chipped vs 226.67±12.7 for females; 308.27±33.65 non-chipped vs 272.91±32.29 chipped males.

### Pathology

During the lifespan of the mice, the general health of the mice was considered normal and no clinical symptoms associated with the implant sites were observed.

On microscopic examination of the implant sites, a thin fibrous capsule was identified in the subcutaneous tissue around a central space where the implant would have been located in 50% of the mice. In two of the 12 animals, small amounts of foreign material, with an associated minimal focal foreign body reaction, was identified in the adipose connective tissue adjacent to the fibrous capsule. These lesions were minor and considered to be of no clinical significance. There was no evidence of generalized or diffuse inflammation, uncontrolled mesenchymal proliferation or tumor formation.

## Conclusion

Insertion of microchips in the groin has no significant effect on the phenotype of mice when assessed using the standard IMPC phenotyping pipeline. While the DEXA results show a significant difference in bone mineral density and bone mineral content between non-chipped and chipped mice, this is to be expected. DEXA relies on transmission of X-rays with high and low energies through a medium to determine the mass per unit (or density) of the medium ^11^; RFID microchips in this scenario being a dense hard material behave as bone and skew the results. The microchip therefore affects the results of this test but not the actual bone mineral content or bone mineral density of the mice. This can be mitigated by cutting out the area of the microchip from the DEXA analysis. However, if microchips are implanted in both test groups and controls this should have no overall effect on the study. Alternatively, the mice can be chipped after the DEXA procedure, but before they recover from the anaesthetic.

The phenotype of an organism is an indicator of how it will function in different environments and under different challenges. Recording the phenotype of a mouse constitutes recording every aspect of clinical, morphological, physiological or cellular change that may arise from any type of experimental manipulation: genetic, surgical or pharmacological ^12, 13^.

Insertion of microchips in the groin requires the application of general anaesthesia; the post-anaesthetic effects of isoflurane on learning and memory may affect behaviour phenotyping ^9, 14– 16^, although this is unlikely with such brief anaesthesia. The current study does not detect any noticeable effects on the phenotype of the mice. The implant does not appear to have any adverse welfare effects on the health of the mice. However, the data is limited to a single inbred wild type strain; genetically modified mice with unknown phenotypes may react differently to the required exposure to anaesthesia.

## Data availability

### Underlying data

Harvard Dataverse: The effects of microchipping C57BL/6N mice on standard phenotyping tests. https://doi.org/10.7910/DVN/PFLBYU ^10^

Data are available under the terms of the Creative Commons Zero "No rights reserved" data waiver (CC0 1.0 Public domain dedication).
