# Supplementary material for: The effects of microchipping C57BL/6N mice on standard phenotyping tests
Source: F1000Res. 2020 Apr 6;9:20. Originally published 2020 Jan 14. [Version 2] doi: 10.12688/f1000research.21633.2 (PMC7194481; doi:10.12688/f1000research.21633.2)
Supplement: Peer review history

# Peer review: Reviewer response for version 2

**DOI:** 10.5256/f1000research.25580.r62041

**Published:** 2020-04-21

**Authors:** Helmut Fuchs, Annemarie Zimprich, Gregor Miller, Manuela Östereicher

**Affiliations:** 1 German Mouse Clinic, Institute of Experimental Genetics, Helmholtz Zentrum München, Neuherberg, Germany; 2 German Mouse Clinic, Institute of Developmental Genetics, Helmholtz Zentrum München, Neuherberg, Germany; 3 German Mouse Clinic, Institute of Experimental Genetics, Helmholtz Zentrum München, Neuherberg, Germany

All our comments to the first version have been completely addressed in the new version.

Is the work clearly and accurately presented and does it cite the current literature?

Yes

If applicable, is the statistical analysis and its interpretation appropriate?

Partly

Are all the source data underlying the results available to ensure full reproducibility?

Yes

Is the study design appropriate and is the work technically sound?

Partly

Are the conclusions drawn adequately supported by the results?

Partly

Are sufficient details of methods and analysis provided to allow replication by others?

Partly

Reviewer Expertise:

Mouse phenotyping

We confirm that we have read this submission and believe that we have an appropriate level of expertise to confirm that it is of an acceptable scientific standard.

# Peer review: Reviewer response for version 1

**DOI:** 10.5256/f1000research.23847.r58613

**Published:** 2020-02-04

**Authors:** Helmut Fuchs, Gregor Miller, Manuela Östereicher, Annemarie Zimprich

**Affiliations:** 1 German Mouse Clinic, Institute of Experimental Genetics, Helmholtz Zentrum München, Neuherberg, Germany; 2 German Mouse Clinic, Institute of Experimental Genetics, Helmholtz Zentrum München, Neuherberg, Germany; 3 German Mouse Clinic, Institute of Developmental Genetics, Helmholtz Zentrum München, Neuherberg, Germany

The manuscript “The effects of microchipping C57BL/6N mice on standard phenotyping tests” by Bains *et al.* describes the comparison of phenotyping data from 12 male and 12 female mice that were microchipped in the groin with unchipped animals (10 males and 10 females). The mice were wild-type C57BL/6N and were analyzed in the IMPC phenotyping pipeline. The experiment is highly interesting, as in the mouse phenotyping community there is a lot of effort put in the development of home cage monitoring systems for an undisturbed observation and analysis of mouse behavior in their normal environment. State of the art technologies require microchipping of the mice for a proper identification of individual mice in the system. Thus, it is of importance to investigate the impact of microchips on other tests that are carried out within the IMPC phenotyping pipeline.

 The experiments and results are well described in the manuscript, however, there remain some questions about the selected methodology and the possible conclusions: we are missing a clear conclusion in the abstract. In the conclusion part there are three statements: “Insertion of microchips in the groin has no significant effect on the phenotype of mice when assessed using the standard IMPC phenotyping pipeline”. “The implant does not appear to have any adverse welfare effects on the health of the mice”. The third conclusion is “The current study does not detect any noticeable effects on the phenotype of the mice”. How far is it via the selected approach possible to obtain information about effects of microchipping on other phenotyping tests – or in other words: how probable is it that there are really no differences if under the given conditions and analysis no differences were detected? For example, the neurological, behavioral and sensory analysis data: The authors decided for an alpha value of 1% and in addition did a correction for multiple testing. Thus, the observed differences need to be very high to reach statistical significance.

 Additional comments:

- Statistical analysis: On which parameters and which tests was the sample size and power calculation based? More detailed information on the power calculation would be desirable. Is there a special reason why the authors select an alpha value of 1%? Also, a more detailed description on the method for multiple testing correction would be helpful.
- Maybe the results part should reflect the order of the tests in the phenotyping pipeline. For a couple of tests from the mentioned phenotyping pipeline there are no results mentioned. For example, in the open-field and SHIRPA analysis the authors report only data from locomotion related parameters. What about the other areas that are covered by these tests (e.g. anxiety and exploratory behaviors)?
- Body weight: According to the provided graphs, at the age of chip implantation (7 weeks) the body weight data flips. In females, non-chipped mice are below, and afterwards are heavier than chipped mice. In males, non-chipped mice are below the chipped mice and then are heavier. Is there any idea about the reason? What is the weight of the RFID? Can the weight of the chip itself make a difference in body weight?
  It might be interesting to know whether the authors also considered other types of RFID’s for the experiment, and why they selected the used one for the study?
- DEXA analysis: Is it possible to cut out the area that is covered by the chip from the DEXA image for the analysis? A DEXA-Image of a mouse with chip compared with an unchipped mouse might be helpful.
  Is there more detailed information available about the material contained inside the chip? If it contains some metal, it might cause scattered radiation that could be harmful for the mouse, and if the DEXA-system is not completely shielded (as it might be the case for older DEXA-systems), also for the experimenter.

Is the work clearly and accurately presented and does it cite the current literature?

Yes

If applicable, is the statistical analysis and its interpretation appropriate?

Partly

Are all the source data underlying the results available to ensure full reproducibility?

Yes

Is the study design appropriate and is the work technically sound?

Partly

Are the conclusions drawn adequately supported by the results?

Partly

Are sufficient details of methods and analysis provided to allow replication by others?

Partly

Reviewer Expertise:

Mouse phenotyping

We confirm that we have read this submission and believe that we have an appropriate level of expertise to confirm that it is of an acceptable scientific standard, however we have significant reservations, as outlined above.

# Response

**Published:** 2020-03-13

**Authors:** Rasneer Sonia Bains

**Affiliations:** MRC Harwell Institute, UK

**Response:** We thank the reviewers for their comments and suggestions on our manuscript. We have tried to address the concerns raised in the responses below and would be amending the manuscript to reflect these changes. 

The manuscript “The effects of microchipping C57BL/6N mice on standard phenotyping tests” by Bains *et al.* describes the comparison of phenotyping data from 12 male and 12 female mice that were microchipped in the groin with unchipped animals (10 males and 10 females). The mice were wild-type C57BL/6N and were analyzed in the IMPC phenotyping pipeline. The experiment is highly interesting, as in the mouse phenotyping community there is a lot of effort put in the development of home cage monitoring systems for an undisturbed observation and analysis of mouse behavior in their normal environment. State of the art technologies require microchipping of the mice for a proper identification of individual mice in the system. Thus, it is of importance to investigate the impact of microchips on other tests that are carried out within the IMPC phenotyping pipeline.

The experiments and results are well described in the manuscript, however, there remain some questions about the selected methodology and the possible conclusions: we are missing a clear conclusion in the abstract.

**Response**: The authors will add the following text in the abstract: “No significant impact of insertion of microchip was observed in any of the phenotyping tests apart from bone mineral density measurement at DEXA due to the nature of the microchip. We therefore recommend that the microchip be inserted during the DEXA procedure, after the measurement is taken but before the   mouse has recovered from the anaesthetic. This would avoid multiple anaesthetic exposures and prevent the potential variability in DEXA analysis output.”

In the conclusion part there are three statements: “Insertion of microchips in the groin has no significant effect on the phenotype of mice when assessed using the standard IMPC phenotyping pipeline”. “The implant does not appear to have any adverse welfare effects on the health of the mice”. The third conclusion is “The current study does not detect any noticeable effects on the phenotype of the mice”. How far is it via the selected approach possible to obtain information about effects of microchipping on other phenotyping tests – or in other words: how probable is it that there are really no differences if under the given conditions and analysis no differences were detected? For example, the neurological, behavioral and sensory analysis data: The authors decided for an alpha value of 1% and in addition did a correction for multiple testing. Thus, the observed differences need to be very high to reach statistical significance.

**Response**: We agree with the concerns raised by the reviewers about the stringent criteria, however, the aim of the current study was to establish the effect of a single procedure on a whole pipeline. We therefore decided to choose the middle ground between using an alpha of 5% and 0.01%, without compromising the integrity of the study.

Additional comments:

- Statistical analysis: On which parameters and which tests was the sample size and power calculation based? More detailed information on the power calculation would be desirable. Is there a special reason why the authors select an alpha value of 1%? Also, a more detailed description on the method for multiple testing correction would be helpful.

**Response**: As discussed in Karp et al., 2015, the calculation of false discovery rate is an ongoing discussion within the consortia. While the current work draws from the ARRIVE guidelines published in Karp et al., 2015, it was felt that as the current study was only carried out in one centre and over a short period of time using an alpha of 0.0001 would perhaps be too stringent and lead to a type 2 error. We therefore chose an alpha value of 1%. Based on an alpha of 0.01 and R=11 tests the alpha was calculated to be 0.001, and not 0.005 as initially reported in the manuscript. We thank the reviewers for picking up this typo, we would like to add here that this does not alter the results. We will correct the text to reflect this.

 

- Maybe the results part should reflect the order of the tests in the phenotyping pipeline. For a couple of tests from the mentioned phenotyping pipeline there are no results mentioned. For example, in the open-field and SHIRPA analysis the authors report only data from locomotion related parameters. What about the other areas that are covered by these tests (e.g. anxiety and exploratory behaviors)?

**Response**: It was not possible, given the character limit to include every parameter in the main body of the text, however all data points have been included in the data repository. Locomotor activity was chosen as the representative parameter as this was the most likely aspect of the mouse behaviour to be affected by insertion of a microchip to the groin. The other biological system most likely to be effected was the metabolism and as such was included in the main body of the text.

 

- Body weight: According to the provided graphs, at the age of chip implantation (7 weeks) the body weight data flips. In females, non-chipped mice are below, and afterwards are heavier than chipped mice. In males, non-chipped mice are below the chipped mice and then are heavier. Is there any idea about the reason? What is the weight of the RFID? Can the weight of the chip itself make a difference in body weight?It might be interesting to know whether the authors also considered other types of RFID’s for the experiment, and why they selected the used one for the study?

**Response**: The weights when expressed as individual data points show a near complete overlap and there is no difference in the two groups in either sex. The slight changes in trend can be attributed to individual animals that have a low in case of females and high starting weight in case of males, which were then randomly assigned to one of the two groups and have therefore continued to influence the trend through the study period.

The RfiDs used in the current study on average weigh approximate 100mg, it is not currently possible to use a wide variety of microchips from different manufacturers as the baseplate readers are tuned to particular chip types and it is not simply a case of swapping one chip for another without tuning and validating the new chips. As the chips used in the current study were used to develop and validate the system we did not deem it necessary to change the chips for the current study.

 

- DEXA analysis: Is it possible to cut out the area that is covered by the chip from the DEXA image for the analysis? A DEXA-Image of a mouse with chip compared with an unchipped mouse might be helpful.Is there more detailed information available about the material contained inside the chip? If it contains some metal, it might cause scattered radiation that could be harmful for the mouse, and if the DEXA-system is not completely shielded (as it might be the case for older DEXA-systems), also for the experimenter.

**Response**: It is possible to analyse the data as suggested, and we will include this suggestion in our conclusion. In addition, we will also include the images as suggested in the supplementary data with the paper.

While the equipment in our laboratories is well shielded and the harm from scattered radiation is very small. The legislation in the U.K. has approved the microchips for use in pets. We agree with the concerns raised and therefore recommend that the mice be chipped after DEXA, before the mice recover from anaesthetic. We will include this in our manuscript.

# Peer review: Reviewer response for version 1

**DOI:** 10.5256/f1000research.23847.r58610

**Published:** 2020-01-23

**Authors:** Jonathan M. Williams

**Affiliations:** 1 Pathobiology and Population Sciences, Royal Veterinary College, North Mymms, UK

In this brief report manuscript, entitled “The effects of microchipping C57BL/6N mice on standard phenotyping tests”, Bains *et al.* describe the development of a home cage analysis system which tracks activity mouse activity through a RFID microchip placed in the groin in 12 male and 12 female c57bl/6 mice at 7 weeks of age.

 This is of particular value as RFID microchips can measure a large number of phenotypic responses with frequent measurements over large time periods without handling or stress inducement in the animal.

 The results demonstrate that importantly, the procedure of chip implantation does not significantly alter across a large number of measurable phenotypic responses - only causing significant changes in measurements of bone density.

 Overall this is a well written report.

 I have only minor criticisms relating to the methods:

- In the husbandry section, it is stated that “While the method of randomisation was simple…”- it is not described. What was the method of randomisation?
- Are animals sedated or under general anaesthesia for chip insertion? Under the microchipping section, it states “This procedure was performed on sedated mice” but elsewhere in the manuscript general anaesthesia is frequently referenced.
- It also states “we have found that a combination of these anaesthetics is the most refined method of delivering these scientific data, as this minimizes any acute discomfort the animal may feel after recovering from the general anaesthetic.”
- There are no details of the manufacturer/source of the RFID chip or software used which would present problems in attempting replication of this experiment.

Is the work clearly and accurately presented and does it cite the current literature?

Yes

If applicable, is the statistical analysis and its interpretation appropriate?

I cannot comment. A qualified statistician is required.

Are all the source data underlying the results available to ensure full reproducibility?

Yes

Is the study design appropriate and is the work technically sound?

Yes

Are the conclusions drawn adequately supported by the results?

Yes

Are sufficient details of methods and analysis provided to allow replication by others?

Partly

Reviewer Expertise:

Veterinary anatomic pathology and intestinal disease

I confirm that I have read this submission and believe that I have an appropriate level of expertise to confirm that it is of an acceptable scientific standard.

# Response

**Published:** 2020-02-03

**Authors:** Rasneer Sonia Bains

**Affiliations:** MRC Harwell Institute, UK

We would like to thank the reviewer for taking the time to review our manuscript and for sharing their comments. Please find below our response to the same.

1.     In the husbandry section, it is stated that “While the method of randomisation was simple…”- it is not described. What was the method of randomisation?

**Response**: The method of randomisation was ‘simple randomisation’, described in the arrive guidelines as, “All animals/samples are simultaneously randomised to the treatment groups without considering any other variable. This strategy is rarely appropriate as it cannot ensure that comparison groups are balanced for factors or covariates that might influence the result of an experiment.”

We will clarify that in the text by rewording the statement to “The method of randomisation used was Simple Randomisation…”

2.     Are animals sedated or under general anaesthesia for chip insertion? Under the microchipping section, it states “This procedure was performed on sedated mice” but elsewhere in the manuscript general anaesthesia is frequently referenced.

It also states “we have found that a combination of these anaesthetics is the most refined method of delivering these scientific data, as this minimizes any acute discomfort the animal may feel after recovering from the general anaesthetic.”

**Response**: The procedure was performed on anaesthetised mice, we will change the text in this section to reflect that.

3.     There are no details of the manufacturer/source of the RFID chip or software used which would present problems in attempting replication of this experiment.

**Response**: The details of the RFID microchips are supplied in the Microchipping section of the manuscript, “These microchips were contained in standard ISO biocompatible glass capsules (11.5×2mm, PeddyMark Ltd. UK).”

The software used to read these RFID data is described in reference 4 - Bains et al., 2016, where the details of the Home Cage Analysis system (Actual Analytics, Edinburgh) have been published. As the RFID data were not part of the current manuscript, the details of the software were not considered essential to the replicate this study.
